# VarDetect: a nucleotide sequence variation exploratory tool

**DOI:** 10.1186/1471-2105-9-S12-S9

**Published:** 2008-12-12

**Authors:** Chumpol Ngamphiw, Supasak Kulawonganunchai, Anunchai Assawamakin, Ekachai Jenwitheesuk, Sissades Tongsima

**Affiliations:** 1 Genome Institute, National Center for Genetic Engineering and Biotechnology, 113 Thailand Science Park, Phaholyothin Road, Klong 1, Klong Luang, Pathumthani 12120, Thailand; 2 Department of Computer Science, School of Engineering and Technology, Asian Institute of Technology, P.O. Box 4, Klong Luang, Pathumthani 12120, Thailand; 3 Division of Medical Genetics, Faculty of Medicine, Siriraj Hospital, Mahidol University, Bangkok 10700, Thailand

## Abstract

**Background:**

Single nucleotide polymorphisms (SNPs) are the most commonly studied units of genetic variation. The discovery of such variation may help to identify causative gene mutations in monogenic diseases and SNPs associated with predisposing genes in complex diseases. Accurate detection of SNPs requires software that can correctly interpret chromatogram signals to nucleotides.

**Results:**

We present VarDetect, a stand-alone nucleotide variation exploratory tool that automatically detects nucleotide variation from fluorescence based chromatogram traces. Accurate SNP base-calling is achieved using pre-calculated peak content ratios, and is enhanced by rules which account for common sequence reading artifacts. The proposed software tool is benchmarked against four other well-known SNP discovery software tools (PolyPhred, novoSNP, Genalys and Mutation Surveyor) using fluorescence based chromatograms from 15 human genes. These chromatograms were obtained from sequencing 16 two-pooled DNA samples; a total of 32 individual DNA samples. In this comparison of automatic SNP detection tools, VarDetect achieved the highest detection efficiency.

**Availability:**

VarDetect is compatible with most major operating systems such as Microsoft Windows, Linux, and Mac OSX. The current version of VarDetect is freely available at .

## Background

Following completion of the human genome project, detection and discovery of single nucleotide polymorphisms (SNPs) is at the forefront of genomic research. The discovery of SNPs may help to identify causative gene mutations in monogenic diseases as well as SNPs associated with predisposing genes in complex diseases [1,2]. Most fluorescence based sequencers produce nucleotide signals (chromatograms) that must be base-called in order to detect the SNP or point mutation. The terms SNP and point mutation are considered synonymous for the algorithm described in this paper. The efficiency of nucleotide variation detection relies mainly on the accuracy of bioinformatic software used to base-call the chromatograms [3-6]. However, most base-calling tools developed for conventional sequencing may not be suitable for SNP detection because they usually misinterpret chromatogram traces at heterozygous base positions. The common sequencing artifacts, which cause most standard base-calling tools to miscall the chromatogram traces, include: 1) polymerase slipage, 2) loss of resolution, 3) contamination and 4) dye blob [7].

SNP discovery would be greatly accelerated if a reliable, automatic SNP discovery tool was available. A commercial automatic SNP detection program called Mutation Surveyor (SoftGenetics) was recently developed utilizing patented anti-correlation technology to increase the efficiency of SNP and mutation detection [8]. Non-commercial programs for SNP detection include PolyPhred (used together with Phred [6,9], Phrap and Consed programs [10]), novoSNP and Genalys. PolyPhred was designed in conjunction with the well-known Phred and Phrap programs to base-call and assemble input chromatograms prior to SNP detection and visualize the results using the Consed program. The current version of PolyPhred is 6.11 beta at the time of writing. The novoSNP program adopts three independent cumulative scores to identify SNPs, and is able to identify more true SNPs (lower false negative rate) than PolyPhred (version 3) [11]. Genalys software attempts to minimize the number of incorrectly assigned (false positive) SNPs by using peak base ratios and surrounding peak information to identify SNPs [12]. Despite the sophistication of the mathematical models widely used in these algorithms, they still report an unacceptably high number of false negative and/or false positive SNPs.

In this study, we present VarDetect, a sequence variation exploratory software to detect SNPs efficiently from fluorescence based chromatogram data. VarDetect supersedes existing automatic SNP detection tools through utilization of rules which account for the common sequence reading artifacts, combined with pre-calculated peak content base ratios. Furthermore, SNPs can be detected by this software using sequencing data obtained from single, or two-pooled DNA samples.

## Results and discussion

**Figure 1:**
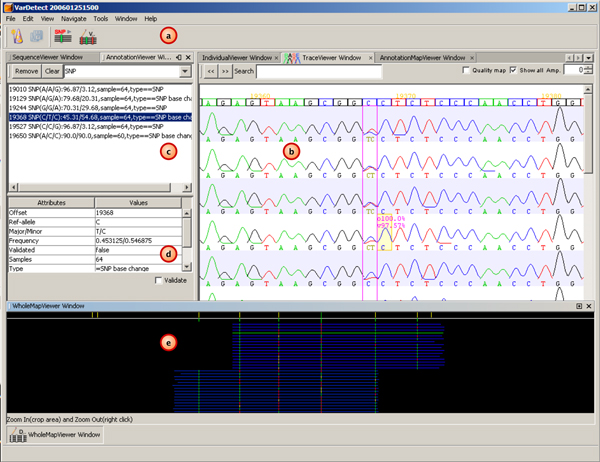
**Illustration of VarDetect's graphical user interface**. The graphical user interface comprises four panes and a quick access toolbar: a) toolbar with a wizard button located on the left-most b) graphical view of input chromatogram traces c) list of predicted SNPs d) SNP information window and e) whole-map view.

VarDetect main graphical user interface (GUI) contains four panes and one quick access toolbar (Figure 1). By pressing the first button on the left of the toolbar (Figure 1a), the program wizard will respectively guide users to 1) load a reference sequence either in an eXtensible Markup Language (XML) or a FASTA format, 2) add input fluorescence based chromatogram traces (.ab1 or .scf extension), and 3) adjust the software parameters such as noise level, heterozygosity level, accepted trace quality and quality difference (*δ*). At the conclusion of these three steps, VarDetect will perform the SNP detection resulting in the graphical traces (Figure 1b), list of predicted SNPs (Figure 1c), detailed information of selected SNPs from the list (Figure 1d), and a whole-map view of the aligned input sequences against the reference sequence (Figure 1e). The whole-map viewer allows users to oversee where the predicted SNPs are located. If the annotated reference sequence (XML format) is used, users can visualize the predicted SNPs to determine if they are novel. As an example, Figure 1e shows the nine SNPs which were previously reported on the reference sequence as short yellow vertical bars on a white horizontal line. The blue horizontal lines indicate the aligned sequences to the reference sequence. The short vertical bars running through these blue lines indicate the predicted SNPs. Furthermore, VarDetect provides an export function that can generate a SNP report using the NCBI SNP submission format.

We compared the features and accuracy of VarDetect with PolyPhred (version 6.11 beta) [10], Genalys (version 3.3.23a) [12], novoSNP (version 2.0.3) [11] and Mutation Surveyor (trial version 3.23) [8]. The efficiencies of these tools were measured in terms of false positive (FP) and false negative (FN) SNP counts. The term true positive count (TP) conveys the number of predicted SNPs which correspond to the actual, prior verified SNPs in the region of interest. The FP is defined as the number of SNPs predicted by the software which are in fact not present. The FN is the number of true SNPs which could not be identified by the software. Hence, the efficiency can be calculated in terms of precision (TP/(TP+FP)) and recall (TP/(TP+FN)) and F-score (2 × (precision × recall)/(precision + recall)) [13]. The performance comparison was made by comparing the results obtained by running these tools against chromatogram traces used in our previous human SNP discovery of fifteen candidate genes. In that report, 171 SNPs were validated from a total of 77 reads [14]. We used these validated SNPs as our TP. Table 1 tabulates the experimental results on the fifteen candidate genes. To avoid *biases *caused by software parameter adjustment, automated detection was performed with the default parameter settings. The detail of these software default parameters are listed at the software website hosted at .

**Table 1:** Comparison of the efficiency of VarDetect, PolyPhred, Genalys, novoSNP and Mutation Surveyor.

**Gene (contigs)**	**Verified SNPs**	**VarDetect**			**PolyPhred**			**Genalys**			**novoSNP**			**Mut. Surveyor**		
		
		TP	FP	FN	TP	FP	FN	TP	FP	FN	TP	FP	FN	TP	FP	FN
*ACOX2 *(5)	10	10	28	0	3	1	7	6	277	4	9	352	1	6	40	4
*ADM *(2)	2	1	4	1	0	0	2	1	260	1	2	220	0	1	11	1
*ARRB1 *(6)	16	15	7	1	9	1	7	16	30	0	15	58	1	13	13	3
*CACNA1D *(11)	26	23	9	3	12	4	14	20	363	6	26	361	0	22	60	4
*CACNB3 *(3)	6	5	4	1	4	1	2	5	191	1	5	308	1	3	51	3
*CCL2 *(2)	3	3	10	0	1	0	2	3	196	0	3	130	0	2	34	1
*CCL3 *(2)	12	11	4	1	4	0	8	9	171	3	12	123	0	9	31	3
*CCL4 *(2)	10	8	11	2	6	3	4	8	136	2	8	96	2	7	20	3
*CCL5 *(2)	3	3	2	0	3	1	0	3	28	0	3	21	0	2	3	1
*CCR7 *(2)	2	2	3	0	1	0	1	2	75	0	2	129	0	1	3	1
*ITGAM *(13)	27	19	20	8	17	7	10	19	552	8	23	618	4	22	63	5
*ITGAX *(15)	25	24	28	1	13	4	12	22	704	3	24	1166	1	19	131	6
*ITGB7 *(9)	16	15	13	1	11	1	5	15	435	1	16	521	0	11	32	5
*LIPG *(1)	4	2	2	2	1	2	3	3	82	1	3	102	1	3	2	1
*NPY *(2)	9	7	4	2	5	0	4	8	228	1	9	334	0	7	11	2

Total 15 genes	171	148	149	23	90	25	81	140	3728	31	160	4539	11	128	505	43
77 contigs																

Precision (%)		49.83			78.26			3.62			3.40			20.22		
Recall (%)		86.55			52.63			81.87			93.57			74.85		
F-score (%)		**63.25**			**62.94**			**6.93**			**6.56**			**31.84**		

Our testing set comprises 77 exonic contigs from 15 human genes. Of these 77 contigs, there are 171 SNPs experimentally validated by the experts [14]. novoSNP has lowest false negative result while generates heighest false positive result (4539). The number of detected SNPs and the false positive result identified by VarDetect are better than Genalys, novoSNP and Mutation Surveyor. Although VarDetect has worse performance on false positive, its SNP identification coverage (148 out of 171) is much higher than that of PolyPhred (90 out of 171) resulting in higher false positive and lower false negative result than PolyPhred' s. From these experiments, VarDetect had the best F-score (63.25%) that slightly greater than PolyPhred (62.94%). Mutation Surveyor, Genalys and novoSNP had low F-score that had 31.84%, 6.93% and 6.56%, respectively.

Of the five tools, VarDetect and novoSNP yielded the lowest false negative counts (23 and 11 respectively), which is of paramount importance in most SNP/mutation discovery projects. PolyPhred reported the fewest false positives (25), while novoSNP reported the most false positives (4539). VarDetect had the second lowest false positive count (149). The chromatograms and analysis results of this experiment can be obtained and visualized online in scalable vector graphics (SVG) format from the above VarDetect website. Finally, we measured the software precision and recall ratios in order to compare them using F-score [13]. These scores are presented in the last row of Table 1 where VarDetect had the highest F-score (63.25%), slightly greater than PolyPhred (62.94%). Despite the very similar efficiency, VarDetect reported considerably fewer FNs (about 3.5 times lower) than PolyPhred; hence, VarDetect is preferable for SNP discovery. This implies higher recall rate (higher sensitivity) which is the ability to detect SNPs even from low or ambiguous chromatogram signals. Nonetheless, higher recall rate has a tradeoff that is having lower precision. In other words, a lot more FPs would be predicted as the program sensitivity is improved. Mutation Surveyor, Genalys and novoSNP had low efficiencies due to much higher numbers of false positives (31.84%, 6.93%, and 6.56%, respectively). Overall, VarDetect is superior to other automatic SNP discovery tools because both FN and FP counts are minimized.

**Figure 2:**
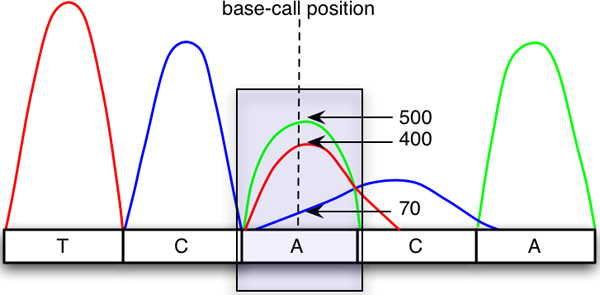
**Chromatogram trace showing peak intensities where dashed line is the base-call position**. Three peaks are detected at this position. The intensities of green, red, and blue peaks are 500, 400, and 70 units, respectively and are used in peak intensity ratio calculation.

**Figure 3:**
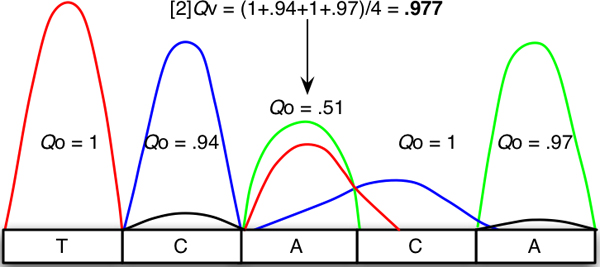
**Calculation of vicinity peak intensity ratio of the base-call position (arrowed)**. [2]-vicinity ratio (k = 2) is calculated by normalizing the surrounding signal intensities of two bases left and right of the observed position as described in Equation 2 as follows: $Q_{v}^{3}$ = 1/4 × (1 + 0.94 + 1 + 0.97) = 0.977

It is worth noting that most FPs picked up by VarDetect in this experiment came from two fundamental problems which are very difficult for the proposed heuristics to resolve. First VarDetect utilizes the vicinity quality concept to implicate how much confidence we can identify a SNP since the quality of the observed base will be bad for both SNP and poor quality signal (see Figures 2 and 3). Because we have preset the software to have a higher recall rate and given that the vicinity quality is good, VarDetect would likely predict the observed base as SNP even with a small trace of turning point. Secondly, VarDetect does not explicitly trim both ends of the input chromatograms which usually contain low quality trace signals. Hence, with current high sensitivity settings, VarDetect tends to predict more FPs. To get around these problems, one must fine tune the parameters, e.g., noise threshold, vicinity/observed quality acceptance etc., to alter the prediction behavior of VarDetect.

Furthermore, we also compared different software features and tabulated them in the feature matrix table (Table 2). VarDetect, Genalys, novoSNP and Mutation Surveyor are more user-friendly since they are not dependent on other external software packages such as Consed, Phrap and Phred. They also offer an embedded graphical user interface while PolyPhred requires an external Consed tool installed to visualize the results. VarDetect and PolyPhred can use batch SNP detection with command line interface (CLI), while the other softwares have only Graphical User Interface (GUI). In terms of processing allele frequencies from two-pooled DNA samples, VarDetect and Genalys are the only two softwares that offer such a feature. Furthermore, VarDetect can read in a reference sequence and corresponding validated SNP locations on the reference sequence to compare with the predicted SNPs, allowing indication of possible novel SNPs. Reference sequences and the corresponding previously reported SNPs can be downloaded directly from the ThaiSNP database [15] in XML format.

**Table 2:** Comparison of the different features between VarDetect, PolyPhred, Genalys, novoSNP and Mutation Surveyor

**List of different features**	**VarDetect**	**PolyPhred**	**Genalys**	**novoSNP**	**Mutation Surveyor**
Operating Systems*	All	All	Windows, Mac	Windows, Linux	Windows
Easy installation	Yes	No	Yes	Yes	Yes
Graphical User Interface (GUI)	Yes	w/Consed	Yes	Yes	Yes
Command line interface (CLI)	Yes	Yes	No	No	No
Allele frequency calculation for	Yes	No	Yes	No	No
two-pooled DNA samples					

(*VarDetect requires Java runtime (JRE) version 1.4 or later)

## Conclusion

We present the framework of a novel algorithm to interpret (base-call) fluorescence based chromatograms and efficiently detect the corresponding nucleotide variations in an automatic fashion. In this framework, three main heuristic procedures are employed: 1) Partitioning and Re-sampling (PnR) algorithm that may be used to base-call the bases with ambiguous signal, 2) calculation of the observed signal intensity ratio(*Q*_*o*_) and vicinity intensity ratio (*Q*_*v*_) and utilizing the differences between *Q*_*v *_and *Q*_*o *_(quality difference) to check whether the heterozygous peaks are correctly called by the PnR algorithm, and 3) conversion of the chromatogram inputs to numeric code using CodeMap so that the variation can be correctly identified by computer.

The experimental results showed that VarDetect is more efficient than other existing tools, namely PolyPhred, novoSNP, Genalys, and Mutation Surveyor for detecting SNPs. VarDetect's heuristics minimize both false positive and negative errors reducing the effort needed to detect and validate SNPs, making it the tool of choice for automatic SNP detection. Furthermore, VarDetect offers the most features including the ability to detect SNPs from pooled DNA samples and the use of XML annotated reference sequence to cross check the SNP discovery results within the tool without using external applications. VarDetect is platform independent since it was implemented in Java, allowing it to run on all major operating systems without recompiling the source codes.

## Methods

After reading the input chromatograms, VarDetect first decides whether or not the chromatogram signal(s) at an observed position is a nucleotide signal by using the intensity determination technique. For each position, VarDetect calculates observed peak content base ratios as follows:

(1) $$Q_{o}^{i}=\frac{\max(\{c^{i}(b)|b\in \{A,T,G,C\}\})}{c^{i}(A)+c^{i}(T)+c^{i}(C)+c^{i}(G)}$$

where $Q_{o}^{i}$ is the signal intensity of a nucleotide at the *i*^th ^position (the base-call position), and is the ratio of the highest signal intensity *c*^*i*^(*b*), where *b *∈ {*A*, *T*, *G*, *C*}, to the sum of the signal intensities of adenine *c*^*i*^(*A*), thymine *c*^*i*^(*T*), cytosine *c*^*i*^(*C*), and guanine *c*^*i*^(*G*), respectively.

Figure 2 depicts a five-peak chromatogram sample. At the indicated base-call position, there are two predominant peaks and one weaker noise signal. Thus the $Q_{o}^{3}$ can be calculated using Equation 1, where the highest signal is 500 (adenine) and the sum of all intensities (*A *= 500, *T *= 400, *C *= 70) at this location is 500 + 400 + 70 = 970. Therefore, $Q_{o}^{3}$ is 500/970 = 0.515. If the middle peak contains only an adenine signal, the ratio would be 500/500 = 1. This calculated ratio can be used to filter out parts of chromatogram that contain low quality peaks. The observed peak content base ratio is then used to calculate an average peak content base ratio of the [*k*]-vicinity bases to the left and [*k*]-vicinity bases to the right of an observed base ([*k*]$Q_{v}^{i}$) which is defined as:

(2) $$[k]Q_{v}^{i}=\frac{1}{2k}\times \sum_{j=1}^{k}\left(Q_{o}^{i-j}+Q_{o}^{i+j}\right)$$

This term is the arithmetic mean of the signal intensities which flank $Q_{o}^{i}$ to the left for *k *bases and to the right for *k *bases. In other words, it is the summation of observed intensities of *k *bases toward the left and right of $Q_{o}^{i}$ divided by 2*k *(Figure 3). The peak content base ratio of the *i*^th ^base from the above definition reflects the changes that occur when peak intensity is altered by having two or more different signals coincident at the *i*^th ^position. Therefore, we can tentatively identify the heterozygous state at the *i*^th ^base by observing the difference between the observed and vicinity peak content base ratios.

**Figure 4:**
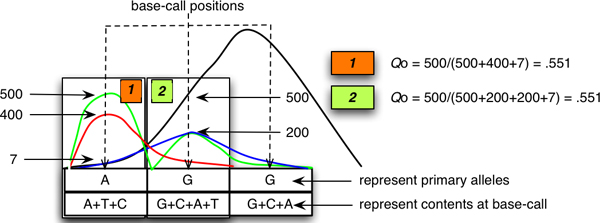
**The peak intensity ratio approach may not correctly base-call different peak patterns**. The *Q*_*o *_value from both boxes 1 and 2 are identical (0.551); however the black peak in box 2 is misinterpreted as a primary peak, since it clearly over-shoots from the adjacent base position.

However, the problem of sampling the values at the *i*^th ^position is that the intensity ratio may be interpreted as being a polymorphic site or just an unreadable nucleotide with noisy signal data. Figure 4 demonstrates that even though the intensity ratios from both nucleotide positions (boxes 1 and 2) are identical, the base-call for box 1 can be made more accurately than box 2. The various signals at box 2 most likely reflect sequence reading artifacts, rather than true sequence polymorphism. VarDetect considers peak shapes in addition to the intensity ratio, which can help to reduce these reading artifacts. Regardless of the nature of the base-calling ambiguity, the concept of intensity ratios for the base under observation and adjacent bases provides a good foundation for the SNP discovery process. The sub-procedures used for automatic SNP discovery and verification comprise the following three steps:

1. Reading nucleotides (base-calling) from chromatogram traces

2. Alignment of input sequences to the reference sequence

3. Detection of SNPs and insertions/deletions (indels)

Each step also comprises a two-fold process, namely rough and fine data processing to ensure the accuracy of the resulting data. Processed data collected from base-calling and trace-alignment are analyzed using the aforementioned intensity ratio concept.

### Reading nucleotides (base-calling) from chromatogram traces

Currently, most chromatogram trace data come in two formats, namely .ab1 and .scf. Although the .ab1 extension format is proprietary, there are numerous bioinformatics tools, e.g., Phred [6,9], PolyPhred [10], 4Peaks [16], FinchTV [17], Genalys [12], novoSNP [11], Mutation Surveyor [8] and software libraries including BioPerl [18,19], BioJava [19], and BioPython [19] that can read this file format. We used Java and BioJava to develop VarDetect because of its high portability. From Equations 1 and 2, the intensity ratios can be pre-computed while base-calling of an input chromatogram is being processed. Each nucleotide position contains intensity values of *A*, *T*, *G*, and *C *as required by the definitions. The algorithm to calculate such ratios strictly follows these definitions.

### Base-calling

**Figure 5:**
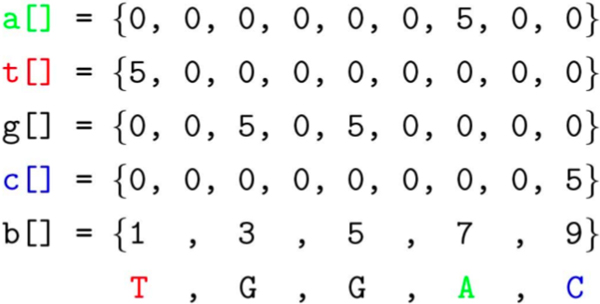
Computer representation (array) of chromatogram traces.

**Figure 6:**
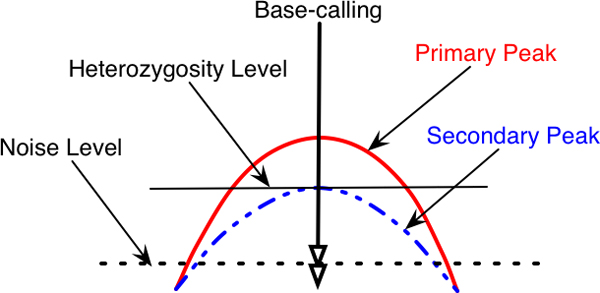
**Base-call parameter setting in VarDetect**. The highest signal is determined as its primary peak, the lower signal is determined as the secondary peak. The signal contents below the noise level are ignored. The heterozygosity level in this setting roughly estimates nucleotide mixture ratio when dealing with pooled DNA.

Base-calling translates the chromatogram traces to one of the four nucleotide characters *A*, *T*, *G*, or *C *along the sequence. When two or more different bases are detected at a calling position, the International Union of Pure and Applied Chemistry (IUPAC) ambiguous nucleotide codes are assigned to that position. At the end of this step, a corresponding sequence is obtained as the base-calling result. By using BioJava, the data from a chromatogram trace is represented by five arrays: base-call array or *B *[], and nucleotide content arrays: *A *[], *T *[], *C *[], and *G *[], for adenine, thymine, cytosine, and guanine, respectively (Figure 5). The base-call array stores the sampling interval where information from the nucleotide content arrays should be read. Figure 5 shows sample array contents which can be read as *TGGAC *by observing the highest amplitude content, 5 in this case, specified by the positions held in the *B *[] array. If more than one nucleotide content array has significant amplitude, the base-call at this position could be interpreted as heterozygous. Base-calling configuration settings such as the noise level and heterozygosity ratio are preset in base-calling programs. These settings are however not optimal for identifying SNPs for all fluorescence based sequencers. Therefore, VarDetect allows users to adjust these values to fit their experiments. Figure 6 depicts the base-call parameters used in VarDetect. The highest signal is assigned as the primary peak, and the lower signal is assigned as the secondary peak. Signal content below the noise level is ignored. The heterozygosity level in this setting roughly estimates the typically observed nucleotide mixture ratio for biallelic DNA. Many base-calling programs misinterpret heterozygous nucleotides as "*N*", or calling error, resulting in false negative SNP detection. VarDetect can accommodate chromatogram input data from mixed DNA samples, which is discussed in detail below.

**Figure 7:**
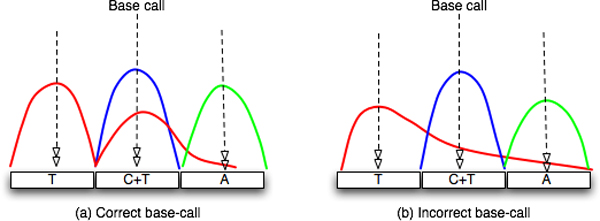
**Effect of signal intensity decay on base-calling**. Correct (a) and Incorrect (b) base-calling interpretation due to signal intensity decay.

Decaying signals from adjacent bases (a frequent reading artifact) can lead to base miscalling. Consider the scenarios in Figure 7. At the base-call position in Figure 7a, both *C *and *T *nucleotide signals are significant in the context of the primary peak, indicative of the true heterozygosity. On the other hand, if part of the primary peak (*T*) from the adjacent base overlaps with the primary peak at the base-call position (*C*) (Figure 7b), the base-calling program may misinterpret this as a heterozygous *C/T *base instead of a homozygous *C *base, with *T *disregarded as background noise. Getting accurate information from input traces is thus crucial for SNP detection. The following section discusses a novel base re-sampling and calling technique, which is an improvement over current base-calling programs.

### Re-sampling and calling

**Figure 8:**
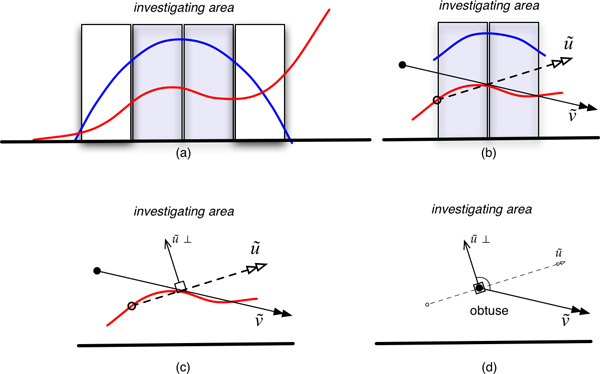
**Improvement of base-calling by using Partitioning and Re-sampling (PnR) technique**. For an observed base (shaded boxes), VarDetect divides a chromatogram peak into four equal parts (partitions) and focuses at the two middle parts (a). The two vectors $\vec{u}$ and $\vec{v}$ are created by connecting the points that the curve segment of the secondary peak crosses over the two partitions (b). Let $\vec{u}$^⊥ ^be a perpendicular vector of $\vec{u}$ by rotating it 90 counter-clockwise (c). Then the secondary peak curve has a turning point if the dot product of $\vec{u}$^⊥^·$\vec{v}$ produces a negative value. In other words, if the angle *θ *between $\vec{v}$ and $\vec{u}$^⊥ ^is obtuse, this secondary peak could be interpreted as being heterozygous peak (d).

**Figure 9:**
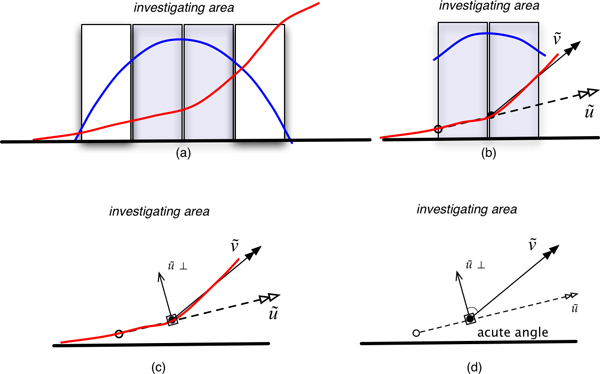
**Illustration of PnR analysis**. Partitioning (a) and Re-sampling (b) of chromatogram with rising (red) peak. $\vec{u}$^⊥ ^is a perpendicular vector of $\vec{u}$ by rotating it 90° counter-clockwise (c). The secondary peak curve has no turning point since the dot product of $\vec{u}$^⊥^·$\vec{v}$. produces a positive value (d). Therefore, this peak is interpreted as a homozygous peak.

In this step, we used the Partitioning and Re-sampling (PnR) technique to improve the base-calling procedure. The main concept of PnR is the detection of secondary peak turning point patterns. VarDetect divides the chromatogram peak at the base-call position into four equal parts (partitions) and focuses on the two central partitions (shaded boxes in Figure 8a). The two vectors $\vec{u}$ and $\vec{v}$ are created by connecting the points that the curve segment of the secondary peak crosses over the two partitions (Figure 8b). Let $\vec{u}$^⊥ ^be a perpendicular vector of *u *by rotating it 90° counter-clockwise (Figure 8c). Then the secondary peak curve has a turning point if the dot product of $\vec{u}$^⊥^·$\vec{v}$ produces a negative value. In other words, if the angle *θ *between $\vec{u}$^⊥ ^and $\vec{v}$ is obtuse, this secondary peak can be interpreted as a heterozygous peak pattern (Figure 8d). In the case of artifactual secondary peak patterns resulting from primary peak overlap from an adjacent base (described above in Figure 7b), the angle between $\vec{u}$^⊥ ^and $\vec{v}$ is acute and PnR would determine this secondary peak amplitude as a decaying event and ignore this signal (Figure 9).

### Alignment of input sequences to the reference sequence

After re-sampling and base-calling, the input sequences are then aligned against the reference sequence using a local alignment method. There are two steps in this alignment process: 1) pre-alignment and 2) alignment enhancement.

### Pre-alignment

Since this tool uses the direct method to search for SNPs, alignment of input sequences to a reference sequence is required. The reference sequence in FASTA format can be obtained from the NCBI public database. VarDetect simplifies the pre-alignment task by linearly searching a local match of *m *contiguous bases greater than or equal to *p *percent. In other words, each individual sequence is aligned to the reference sequence by sliding a window of *m *adjacent bases (*W*_*m*_) along the reference sequence until a match of *p *percent or greater is found. Since the noisy parts of input chromatograms can be filltered out using the intensity peak ratio concept explained previously, the candidate window *W*_*m *_is selected from good intensity areas of the input chromatogram. From this observation, we investigate the vicinity peak ratio such that [*k*] ≥ 90%, starting from the position *i *≥ (0.2 × *N*), where *N *is the total number of peaks (or 20% of the total number of bases).

The 90% quality value is used to guarantee that the selected regions are readable and good enough for automatic SNP detection. The *W*_*m *_window is formed by inclusively extending the next *m *bases from the accepted observed base *i*. The *W*_*m *_window is chosen for each chromatogram trace based on this selection scheme. Each trace is aligned with the reference sequence using *W*_*m *_as its representative in matching the pattern whose percent similarity is greater than a given value. This algorithm, called "Quick Alignment using Sliding Window", is applied repeatedly to more than one *W*_*m *_window to optimize the alignment. The alignment process is performed on both forward and reverse orientations and applied iteratively to each chromatogram.

### Alignment enhancement

**Figure 10:**
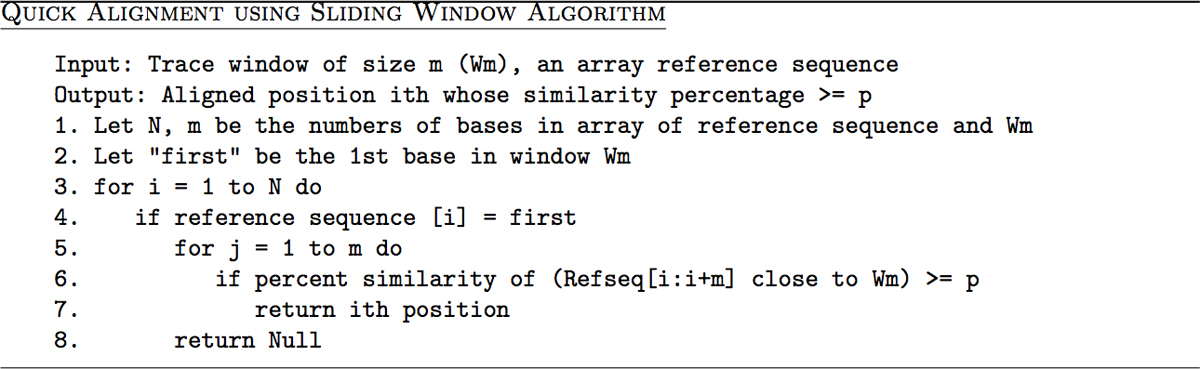
Quick alignment using sliding window algorithm.

VarDetect uses a sliding window *W*_*m *_to get several local alignment candidate fragments. To complete the overall alignment task, VarDetect increases the window size of each candidate fragment by extending the base-matching from both ends of the window. The longest matching result is then selected. The algorithm is described in Figure 10

### SNP selection

SNPs can be in two forms: homozygous and heterozygous. The homozygous form can be detected easily by comparison with the reference sequence, while the heterozygous form can be detected by observing differences between *Q*_*v *_and *Q*_*o*_. For true SNPs, there are two dominant nucleotides, which result in a low observed intensity ratio *Q*_*o *_(Equation 2). Therefore, significant differences between *Q*_*v *_and *Q*_*o *_are indicative of SNPs. This value *δ *= *Q*_*v *_- *Q*_*o*_, called the detection value, is extensively used to mask out non-SNP regions.

However, inappropriate setting of the *δ *value may lead to wrong SNP identification. If *δ *is too low, the number of false positives would be high, since a slight drop of peak height could be detected as a mutation. Conversely, if *δ *is too high, the number of false negatives is high. The *δ *value should be adjusted prior to performing automatic SNP detection since this value may differ among experimental protocols. From our empirical study results, the default (optimum) value should be set to 12.5%.

SNP detection is most accurate when analyzing sequence data obtained from individuals, since homozygous bases have a single chromatogram peak, and heterozygous bases have two peaks of similar intensities. Recently, it has been proposed that pooling of DNA samples from more than one individual can accelerate and reduce the cost for SNP discovery. However, there is the limitation that different DNA samples will be sequenced with different sequencing reaction efficiencies, owing to variable DNA quality and concentration, and variable affinity of DNA polymerase for different nucleotides [12,14]. Despite this limitation, VarDetect can still accurately calculate allele frequencies and detect SNPs from chromatogram traces derived from pooled DNA samples.

When DNA samples from two individuals are pooled, there are five possible biallelic combinations, each producing different chromatogram patterns (Table 3). At a given base *i*, if both individuals are homozygous (biallelic combinations XXXX and YYYY) or heterozygous (XXYY), the primary and secondary peak intensities would not be different from the data obtained from single samples analyzed separately. On the other hand, if one individual is heterozygous, while the other homozygous (XXXY and XYYY), a weak secondary peak would be observed which must be accounted for by the SNP detection algorithm, or otherwise that SNP would be missed. For this situation, the *δ *term in VarDetect approximates the amplitude of the secondary peak *i*, since both *Q*_*v *_and *Q*_*o *_are obtained from the ratio of primary intensity signals normalized to the sum of all signals.

**Table 3:** Five possible biallelic outcomes of sequencing two-pooled DNA samples

No.	Scenarios	Peak Content		Fusion/Combination
			
		primary	secondary	
1	XXXX	4	0	(XX)+(XX)
2	YYYY	4	0	(YY)+(YY)
3	XXYY	2	2	(XX)+(YY)-or-(XY)+(XY)
4	XXXY	3	1	(XX)+(XY)
5	YYYX	3	1	(YY)+(XY)

### CodeMap

**Figure 11:**
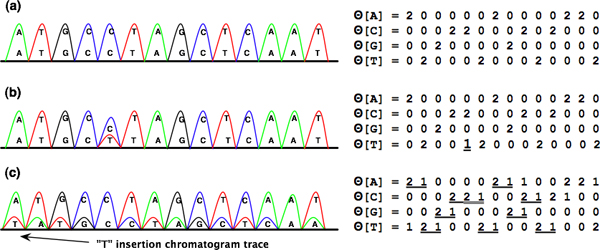
**Illustration of traces with indels and their CodeMap analysis**. Noise-eliminated homozygous (a), homozygous with a *C/T *SNP at the 5^th ^position (b), and *T *insertion at the first position (c) chromatogram traces.

After the re-sampling and calling procedure is completed, accurate base-calling can be done on the chromatogram while noise which may interfere with SNP detection is removed. This "processed" chromatogram is then converted into a numeric code via our CodeMap technique prior to SNP and indel detection in VarDetect. This section describes the generation and interpretation of the code. Figure 11 shows examples of a homozygous chromatogram trace (Figure 11a), a chromatogram with one *C*/*T *SNP at the fifth base (Figure 11b), and a chromatogram with *T*-insertion at the first base (Figure 11c). CodeMap individually observes variations associated with *A*, *C*, *G *or *T *and performs the conversion from the biallelic values at each base position to 0, 1, and 2 according to the following rule. Two prominent values, primary and secondary peak intensities, at each base *i*^th ^are symbolized as $b_{\text{pri}}^{i}$ and $b_{\text{sec}}^{i}$ respectively.

Four code sequences Θ[*A*], Θ[*C*], Θ[*G*], and Θ[*T*] collectively termed Θ[*N*], where *N *represents either *A*, *C*, *G *or *T*, can be constructed by:

(3) $$\begin{array}{cc} \Theta [N], & \text{base }b^{i}=\{\begin{array}{ll} 2 & \text{if }N\text{ is }b_{\text{pri}}^{i}\\ 1 & \text{if }N\text{ is }b_{\sec}^{i}\\ 0 & \text{if }N\text{ is not }b^{i} \end{array} \end{array}$$

Using Equation 3, CodeMap converts the chromatograms in Figures 11 to numeric arrays. The homozygous base is converted into 0 and 2 codes (Figure 11a) while heterozygous base in Figure 11b (Θ[*T*]) is converted to 1. VarDetect can make use of this code in conjunction with the aforementioned *δ *value to automatically detect SNPs.

In addition to identifying nucleotide substitutions, VarDetect also automatically detects indels through CodeMap (Figure 11c and its corresponding numeric arrays). When one base (*T*) is inserted, the following bases are shifted by one frame to the right. Such indels cause misinterpretation errors in most base-calling approaches. To overcome this problem, the indel chromatograms have to be manually edited by skilled operators.

In the CodeMap view, the code sequence which one would expect to often see from this phenomenon is 2(1/2), the code 2 following with either 1 or 2. With an observation on any list in base array (Θ[*N*]), CodeMap generates this insertion pattern by isolating the correlation between the shifted bases with each possible nucleotide type that is eventually identified by VarDetect. For the adenine array (Θ[*A*]) in Figure 11c, there are two patterns of "2 1" or "2 2", with four counts, "2 1", "2 1", "2 2", and "2 1". Pattern "2 1" is derived from an observed base *A *(represented by code 2) being shifted by one position whose base content is not an adenine; hence, it appears in the next position as a heterozygous peak (represented by code 1). Pattern "2 2" is derived from an observed base *A *(represented by code 2), which is shifted by one position whose content is an adenine. Thus, the next position becomes homozygous *A *(represented by code 2). These patterns also occur in the cytosine (Θ[*C*]), guanine (Θ[*G*]), and thymine (Θ[*T*]) arrays. For one base deletion, the pattern to be detected is reversed to (1/2)2, the code 1 or 2 following with code 2.

The CodeMap principle may be applied to detect more than one base indel. One would expect to see the pattern 2?[*k*](1/2) code 2 following with *k *number of 0, 1 or 2 (we use "?[*k*]" to represent them) and then finishing with either code 1 or 2. For example, when bases *CG *are inserted in front of the sequence, the pattern 2?[1](1/2) can be observed. In this data set, other patterns are also possible: 2(1/2) (one base insertion), (1/2)2 (one base deletion), 2?[2](1/2) (three bases insertion) and others (Table 4). In general, *N*-base indel patterns are generated by a *N *- 1 combination of 0, 1, and 2 codes. Therefore, there are multiple patterns possible in each base array. VarDetect simply counts the number of occurrences of each pattern and uses the patterns with the highest frequencies to determine indel types. Table 4 illustrates the pattern counting from Figure 11c. Here the dominant pattern with 13 occurrences is 2(1/2). This CodeMap technique along with pattern detection can be used to efficiently detect SNPs and indels automatically.

**Table 4:** Pattern counting of numeric code shown in Figure 11c. VarDetect selects the highest frequencies (13) to determine number of indel bases.

	Pattern Counts					
Pattern	Θ[*A*]	Θ[*C*]	Θ[*G*]	Θ[*T*]	Total	Comment

2(1/2)	4	4	2	3	13	1 insertion
2?[1](1/2)	1	2	0	0	3	2 insertion
2?[2](1/2)	0	1	0	0	1	3 insertion

(1/2)2	1	2	0	1	4	1 deletion
(1/2)?[1]2	0	1	0	0	1	2 deletion
(1/2)?[2]2	1	1	0	3	5	3 deletion

**Figure 12:**
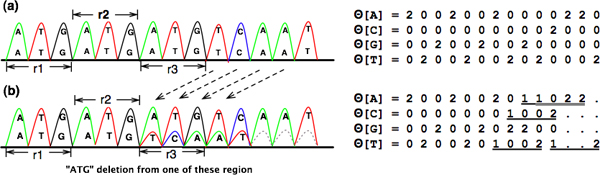
**Illustration of VNTR with ATG deletion and its CodeMap analysis**. CodeMap converts chromatogram of trinucleotide repeats (*r*_1_, *r*_2_, *r*_3_) *to the corresponding numeric arrays *(2 0 0 2 0 0) on the right (a). When a set of trinucleotide repeats is deleted, CodeMap reveals specific numeric patterns (underlined) on the right (b), which match with (1/2)?[2]2 pattern shown in Table 4.

Furthermore, for simple indels found in variable number of tandem repeats (VNTR), where the repeat unit composed of more than one nucleotide can increase or decrease in number within a tandem repeat array in the sample sequence, we can theoretically apply Codemap to detect such a case (Figure 12).

## Competing interests

The authors declare that they have no competing interests.

## Authors' contributions

ST and SK came up with the idea to create VarDetect. SK developed the software while each subroutine in VarDetect was conceived by AA, CN and ST. ST obtained funding for this project. Finally, ST and EJ drafted this manuscript. Note that CN, SK and AA are regarded as equally contributing first authors.
